# Contemporary pedagogy? The use of theory in practice: An evidence-informed perspective

**DOI:** 10.3389/fspor.2023.1113564

**Published:** 2023-03-21

**Authors:** Robin D. Taylor, Jamie Taylor, Michael Ashford, Rosie Collins

**Affiliations:** ^1^ School of Health and Human Performance, Faculty of Science and Health, Dublin City University, Dublin Ireland; ^2^ Grey Matters Performance Ltd., Stratford upon Avon, England; ^3^ Insight SFI Centre for Data Analytics, Dublin City University, Dublin Ireland; ^4^ Moray House School of Education and Sport, The University of Edinburgh, Edinburgh, Scotland

**Keywords:** coaching, cognitive load, desirable difficulty, expertise, fidelity, pedagogy, professional judgement and decision-making

## Abstract

Utilizing cognitive psychology as a foundation, this paper offers a deeper consideration of contemporary theoretical influences on coaching pedagogy. Countering recent dichotomies suggested between pedagogic approaches, we reintroduce key findings from the cognitive tradition and their implications for practice which coaches may find useful. Using cognitive load, novice and expert differences, desirable difficulty, and fidelity, we suggest that the lines drawn between different “pedagogies” may not be as sharp as suggested. Instead, we suggest that coaches avoid defining themselves as being aligned to a specific pedagogical or paradigmatic stance. We conclude by advocating for research informed practice, absent of strict theoretical boundaries and instead, considering contemporary pedagogy as drawing on the needs of the context, the experience of the coach and the best available evidence.

## Introduction

Across all levels of sport, the role of the coach is to support athletes to acquire, develop and enhance sporting (and non-sporting) attributes, skills and understanding (1). Consequently, to meet a range of participant performance and development needs [cf. (2)], coaches must develop a breadth of pedagogic understanding across instruction methods and learning design (3). Coaches, therefore, require a strong knowledge, understanding and application of sport pedagogy to shape their environment appropriately [cf. (4)]. Acknowledging the complexity of coaching practice, Nash and Collins (5) identified three bodies of knowledge necessary for coaches to be effective in their practice: sport specific, the ‘ologies and pedagogical knowledge, with pedagogy being defined as “any conscious activity by one person designed to enhance learning in another” [p. 3 (6)].

Despite this necessity and the practical need, pedagogy has tended to be marginalized across research and coach education [cf. (1, 7, 8)]. Indeed, pedagogic research in coaching has tended to be conducted from foundations of behaviorist (1, 9), cognitivist (10), constructivist (1, 9), critical realist (11) and ecological (12) perspectives. In some cases, these theoretical foundations have driven practical implications based on epistemological alignment. Thus, there is a need for coaches, practitioners and researchers alike (in their pursuit of enhanced coaching practice) to navigate the differences between theoretical stances in order to progress towards practical utility for coaches (1).

Exemplifying this, recent literature has seen a significant growth of skill acquisition as a pedagogical domain of study. Concurrently, differences in theoretical foundations have led to a domain specific dichotomy between pedagogical tools deriving from Information Processing (IP), such as contextual interference within practice design [cf. (13)], and Ecological Dynamics (ED) and the resultant principles of non-linear pedagogy [cf. (14)]. The question of whether other literature has moved beyond this delineation notwithstanding [cf. (15)], this, perhaps false, dichotomy has spilled into the pedagogic domain.

Recently, a range of papers have discussed the notions of contemporary pedagogy, one that is underpinned by ED and in contrast to a “traditional pedagogy” based on earlier learning theories such as cognitivism, constructivism and pedagogical theories of instruction [e.g., (16–19)]. From this perspective athlete learning, and subsequent pedagogical implications center around the mutuality of the participant and environments, taking into consideration complexity and variability (20). Whilst we acknowledge the utility of nonlinear pedagogical tools in practice [cf. (21)], here we challenge the notion of a “traditional” vs. “contemporary” pedagogy. Our suggestion is that a continued acceptance of a false dichotomy has the potential to unnecessarily divide opinion, and in turn, hinder the practical development of our field. Other false dichotomies have a long history of critique both in education [e.g., (22)] and in the coaching literature (11, 23–26). Thus, the creation of a traditional vs. contemporary divide has led to researchers and subsequently, coaches who characterise pedagogic interventions under a single theoretical paradigm. For example, authors have aligned cognitive theory with the suggestion that all skills are learned through the reproduction of “the correct” technique through coach led prescriptive instructions and corrective feedback, a view which is misleading and inaccurate (2).

Thus, the purpose of this paper is not an attempt to advocate for individual pedagogic stances over others, nor to challenge the utility of any particular perspective. Instead, by highlighting elements of cognitivist learning theories, we aim to challenge their association with, and pejorative use of, the term “traditional” pedagogy [cf. (27)]. In turn, we discuss how we might progress from the notion of “contemporary”, to evidence-informed pedagogy [e.g., (26)]. Consequently, this paper has two aims, firstly, we reintroduce several key concepts from cognitive psychology and, in turn challenge notions of a sharp pedagogic dividing line. Secondly, we outline pedagogic implications to move beyond notions of either/or and instead focus on nuance in application.

## Cognitive load theory

One of the most prominent theories emerging from cognitive educational psychology is cognitive load theory (CLT). A core feature of CLT is the emphasis on prior learning as shaping the ability of the participant to engage with coaching (28). In essence, what a participant knows, perceives and understands prior to a learning activity or instruction will directly influence the way they learn (26). Despite this, recent ideas within sports coaching research have moved towards methods which promote discovery learning (29) and learning through experience (30, 31). Unfortunately, this shift has overshadowed a significant body of research exploring the relationship between prior knowledge, the presentation of new knowledge and how learning happens. Sweller (32, 33) introduced the concept of CLT as an explanatory theory, blending the notion of human cognitive architecture and instructional consequences. The theory captures the interaction of long term memory and working memory, defining learning as a “change in long-term memory” [p. 39 (34)].

In contrast to the lay use of the term, Sweller (34) suggested that a participants' long-term memory is formed of neural representations [cf. (35)], permitting adequate solutions to problems perceived in our environment (36). These neural representations develop incrementally and are largely obtained by observing, reading and imitating other forms of knowledge (34). Subsequently, the acquisition of knowledge and understanding can be seen as almost entirely inherited from others' long-term memories. When participants are presented with novel information and new ways of thinking/behaving, a load is placed on working memory, which is limited in terms of capacity and duration (37). As guidance, an adult can hold seven new pieces of information, but only process meaning from four, highlighting its limitations (38).

From a coaching perspective, it is essential to consider how learning design and coaching approach can impact the different forms of cognitive load; intrinsic and extraneous (34). Intrinsic load is that which is imposed by the movement to be learned, or the information needed to achieve goals. Extraneous load refers to unnecessary load that is extraneous to learning goals, most often the result of a coach's approach, or their learning design. Both intrinsic and extraneous load are managed by working memory resources. Those resources devoted to what is relevant to learning are considered germane (38). Hence, germane resources are not imposed by learning tasks, but refer to the cognitive resources devoted to developing representations as an adaptation from the working to the long-term memory (39). Combined, these equate to a participant's overall cognitive load (40).

## Desirable difficulty

Cognitive psychology has historically and repeatedly emphasized that effortless learning is ineffective (41). A reduction in extraneous cognitive load does not suggest that the participant experience should be easy or effortless (42). Instead, slowing down, perturbing and challenging performance has the potential to generate, long-term learning (43). One of the most robust constructs emanating from cognitive psychology is the notion of desirable difficulty, defined as the introduction of manageable challenges into the learning environment (43, 44). In motor learning this is referred to as contextual interference. Magill (45) suggested higher contextual interference in practice leads to enhanced motor learning when tested through later retention or transfer. However, key to the effective use of desirable difficulty is consideration of the desirability of any challenge built into practice. Such difficulty must be manageable with the aim of what Bjork [p. 199 (42)] referred to as creating “meaningful rather than misleading” experiences for the participant. In essence, coherent with CLT, if the participant lacks the knowledge or skills to respond to the challenge, difficulties are no longer desirable (44). Therefore, successfully introducing difficulty into the learning environment requires constant monitoring of participant competence and progress (43).

## Expertise/novice differences

This notion of what is desirably difficult is based on the needs and stage of the participant. In understanding the needs of participants, a range of key theories, such as Fitts and Posner's (46) stages of learning, or Gentile's (47) two stage model of development, have attempted to explain the progression from novice to expert within skill acquisition and execution. Motorically, the novice stage is characterized by inconsistent and often clunky performances, leading to much smoother and more controlled performances at expert stage. Pertinently, however, progression is the common feature of these theories. Indeed, becoming expert is a deliberate process of learning, not something which happens to us. Eccles (48) summarizes this when referring to the nature/nurture debate in the development of expertise, identifying that the contribution of expertise development of domain specific abilities achieved through interventions such as deliberate practice (49) outweighs that of a genetic basis for the same abilities. Therefore, to better understand how to develop expertise, researchers have attempted to identify the behavioral characteristics demonstrated by experts, confirming these comparisons with novice counterparts. For example, Chase and Simon's (50) research identified that expert chess players' recognition of patterns of offensive and defensive positions and the anticipation of future possibilities, significantly exceeded that of novices. Fundamentally, experts' long-term memory allowed for higher performance than novices, having been developed over time.

Expanding this, across multiple performance domains, research has attempted to identify features which typify expertise. These include features such as speed with which decisions are made, in that experts may be slower at first to solve a problem when compared to novices but will be quicker overall (51), or that experts will typically take more information, or deeper meaning, from cues (5). However, this does not always lead to the best performance outcome, as experts are more negatively impacted than lesser skilled performers when faced with incongruent information (40). Finally, storage of information whereby experts are able to recall information with more ease than their non-expert counterparts, perhaps due to the hierarchical structure of information storage (52).

## Fidelity

The final area for consideration is the notion of fidelity of practice. Highlighting the complementarity of theoretical positions, fidelity has roots in behaviorism [e.g., (53)] and has subsequently been emphasized by ED in the form of representative practice design [e.g., (54)]. In addition, fidelity of practice has been a consistent focus for cognitive scholars and coaching literature. For example, whilst not explicitly linked with cognitive theory, practice fidelity was emphasized in the work of the English Football Association's Director of Coaching Allen Wade, who suggested that “all coaching should begin with some form of realistic competitive situation” and that lower levels of fidelity should only be used when “working for a clear understanding of ideas” [p. 186 (55)]. Building on this perspective, pedagogic literature began to advocate for the use of games-based approaches in coaching. For example, Teaching Games for Understanding (56) and Game Sense (57) were grounded in features of cognitivist thinking (58). Whilst it would be an oversimplification to suggest that these approaches only advocated for high physical fidelity, a key feature has been that “individual performance must be contextualized in the game” [p. 491 (59)]. In essence, there has been a consistent advocacy for game form practice for a number of decades (60)^1^.

This pedagogic emphasis has been mirrored by the cognitive literature in motor learning with the specificity of practice being consistently emphasized (61). Theories in this area suggest that learning is enhanced under conditions of practice that mirror those required by the task to be learned (62), that: “the amount of transfer obtained between situations is a function of the perceived similarity” [p. 39 (63)]. Secondly, greater perceived similarity between training and performance task triggers the retrieval of mental representation, leading to increased likelihood of transfer (64). This suggests that it may not be just maximizing physical fidelity that is important for learning (65). For this reason, other “types” of fidelity have been suggested such as psychological and conceptual, the former referring to the extent to which “the training environment prompts the essential underlying psychological processes relevant to key performance characteristics” [p. 76 (66)]. In addition, similar to the notion of “exaggeration” in games-based coaching [e.g. (67),], it has been recommended that “conditions being imposed on games emphasize a particular technique” [p. 188 (55)], bearing a strong procedural resemblance to the manipulation of constraints in practice. This emphasis on the manipulation of “types” of fidelity has continued with a recent conceptual problem-solving emphasis (68). A consistent research finding being that learning is supported by the provision of the minimum necessary level of fidelity, based on participant needs, to reduce extraneous cognitive load (69). More recent literature continues to grapple with the nuance of these ideas. For example, findings that suggest that situation-specific contextual information and anxiety have a significant effect on perceptual-motor skill (40). The consequent recommendation being the need for coaches to develop practice conditions that take account of different types of fidelity to optimize learning.

## Implications for practice

Building from these concepts and to further challenge sharp dichotomies, here we outline evidence-informed implications. Thus, illustrative of meaning, we suggest three general pedagogic implications;

i) The necessity for valid assessment of prior knowledge and ability (39)
ii) A consideration of pedagogic methods designed to maximize the resources devoted to what needs to be learned, rather than extraneous factors (34)
iii) Tracking learning over time (70)

In this sense, learning is not *always* seen as resulting from engagement, but instead a process that can and should be planned for and orchestrated by the coach. For example, the “extended challenge based framework for practice design in sport” (71) suggests the need to create meaningful difficulties that are specific to the demands of competition to maximize transfer. The design of meaningful difficulties will require the coach to understand the prior knowledge and ability of participants, relative to the focus of the session. This will aid in identifying what the requirements are for the participants (72). Once this is identified, there is a need to consider the “content” to be learned [the sport specific demands, (72)], the level of participant and the desired learning experience. With difficulty in practice conditions being a function of the relationship between the “nominal” task difficulty (i.e., the constant amount of task difficulty regardless of the performer or performance) and “functional” task difficulty (i.e., how challenging the task is relative to the performer's skill and the performance), practice should change as the participant's level of skill changes (73). These desired difficulties will also be shaped by a coach's approach and learning design, both of which should aim to manipulate intrinsic load appropriately (34). Learning design should consider the extent and type of fidelity that the participants might desirably be exposed to. It may be the case that the coach should emphasize different types of fidelity (physical, psychological, conceptual) for different learning outcomes (66, 69). It would also suggest that tasks should be designed without extensive rules or constraints that might impose significant extraneous load. Furthermore, Table 1. highlights some important cognitive load effects relevant to coaching when considering the design of appropriate practices with any given group.

**Table 1:** Cognitive load effects, evidence and practical implications in sports coaching (adapted from Sweller, 2010).

Cognitive load effect	Description	Type of CL	Evidence within sport coaching domain
Worked-examples	Demonstrations, vicarious experiences, video footage of successful examples—results in better performance when solving problems.	Extraneous	Hodges and Franks (2002) Richards, Collins & Mascerenhas (2012)
Completion	Providing partially solved problems (guidance) rather than full problems (discovery) results in better performance	Extraneous	Hodges & Lohse (2022) Cope & Cushion (2020)
Redundancy	Presence of sources of information that do not contribute to acquisition or automation	Extraneous	Furley & Memmert (2012)
Expertise reversal	Coaching methods that are effective with novices are ineffective with experts and vice versa	Extraneous	Williams & Hodges (2005) Porter & Mcgill (2010)
Guidance fading	As knowledge and understanding increases guidance should be removed i.e. completion problems, then full problems	Extraneous	Pill et al (2021) Ashford et al (2022)
Variable examples	Providing examples with variable features enhance learning compared to examples with similar features	Germane	Pesce et al (2016) Carson et al (2014)
Imagination	Imagining successful completion of problems will enhance learning compared with studying	Germane	Kraeutner et al (2016)
Element interactivity	Cognitive load effects are only obtainable when the elements being learned are interactive	Intrinsic	Healy & Wohldmann (2012)
Isolated / interacting	Present new information in an isolated fashion first, then in an interacting fashion following	Intrinsic	Hodges & Lohse (2022) Carson et al (2016)

Similarly, a coach's approach to practice should also be based on desirable learner experience. Here, more direct approaches may be appropriate for the early stages of learning, or when learning design promotes a significant intrinsic load, a more direct approach is likely appropriate [e.g., instruction, (26)]. Later, as the target knowledge or skill is developed, less guidance is likely more appropriate (74, 75). For example, novices may benefit more from more isolated practices to facilitate “getting the idea of the movement” [p. 334 (76)], alongside more instruction (77), allowing for early opportunities for error correction and movement exploration, before progressing to more variable practice (76). With practice variability potentially overloading until the participant grasps the basic dynamics of the task and can replicate an initial motor pattern (78). Intermediate participants may benefit from a more random schedule of practice, encouraging the participant to retrieve and organize the required actions on each task (79) or the consideration of worked examples [e.g., two vs. one, (77)]. Finally, participants with a higher level of expertise may benefit from greater contextual interference, and pedagogic strategies such as problem solving and guided-discovery (77). Note, however, that this does not necessarily refer to the overall level of the participant, it instead refers to their ability in the target skill. Crucially, managing the level of task difficulty and desired difficulty has a significant impact on participant motivation (80).

## Conclusion

In concluding, our aim throughout this paper has been to offer a perspective on the issue of “contemporary” practice in sport pedagogy and to gently challenge conceptions of practice that seem to associate cognitive theories with a very particular form of learning design that may be “traditional” in some settings. For us as practitioners and pragmatic researchers, we are interested in the value of knowledge for practice and the difference it makes (81). For this reason, we need to remain open to a range of different perspectives, understanding where theories “best fit”. None of this is to suggest that the pedagogies underpinned by the ED perspective offer nothing new, especially given the emphasis on self-organization as the most appropriate means of development (16). Nor is it to say that the constraints led approach or nonlinear pedagogy do not offer value for coaches. They have and will continue to make a significant impact on coaching.

Furthermore, whilst this perspective article highlights constructs developed through cognitive psychology, we by no means suggest that they present answers to all the pedagogic problems we face. Our aim is to progress beyond oversimplifications such as notions of a “contemporary pedagogy”. This is especially important with the growth of alternative representational theories beyond those of the cognitive tradition [e.g., Active Inference, (35, 82)]. As these stances evolve our understanding of human functioning, it will be important to recognize the contribution of previous theory to practice. We should of course discard concepts that do not stand up to scrutiny, but wholesale pedagogic revolution is unlikely. As such, we urge practitioners to avoid defining themselves based on a single theory or methodology. If it is really a question of picking a particular approach, in the absence of overwhelming evidence, this would seem to present the coaches with an impossible choice. One that requires an ideological, rather than evidence-informed perspective. Therefore, rather than an attachment to a specific paradigm, what is “contemporary” pedagogy *should* be what works for those we are coaching, based on the needs of the context and the best available evidence; the very essence of evidence-informed practice (83).

## Data Availability

The original contributions presented in the study are included in the article/Supplementary Material, further inquiries can be directed to the corresponding author/s.

## Footnotes

^1^We refer the interested reader to the excellent summary in Pill (2020)
